# Statement of Retraction: Deciphering cell–cell interactions and communication in the tumor microenvironment and unraveling intratumoral genetic heterogeneity via single-cell genomic sequencing

**DOI:** 10.1080/21655979.2026.2641945

**Published:** 2026-03-12

We, the Editors and Publisher of the journal *Bioengineered*, have retracted the following article.

Cao, Y. H., Ding, J., Tang, Q. H., Zhang, J., Huang, Z. Y., Tang, X. M., … Fu, D. (2022). Deciphering cell–cell interactions and communication in the tumor microenvironment and unraveling intratumoral genetic heterogeneity via single-cell genomic sequencing. Bioengineered, 13(7–12), 14974–14986. https://doi.org/10.1080/21655979.2023.2185434

Following publication, internal integrity audits revealed concerns around several authorship changes which were made between submission and acceptance of the article. 

We have contacted the authors for an explanation, but have not received a satisfactory explanation regarding the contributions of authors added or removed at different stages and the rationale for these changes. As determining authorship and contributions is core to the integrity and accountability of published work, we are therefore retracting the article. The authors have been informed of this decision.

We have been informed in our decision-making by our policy on publishing ethics and integrity and COPE guidelines. 

The retracted article will remain online to maintain the scholarly record and will be digitally watermarked on each page as ‘Retracted’. 

